# Bypassing P-Glycoprotein Drug Efflux Mechanisms: Possible Applications in Pharmacoresistant Schizophrenia Therapy

**DOI:** 10.1155/2015/484963

**Published:** 2015-09-27

**Authors:** Famida G. Hoosain, Yahya E. Choonara, Lomas K. Tomar, Pradeep Kumar, Charu Tyagi, Lisa C. du Toit, Viness Pillay

**Affiliations:** Wits Advanced Drug Delivery Platform Research Unit, Department of Pharmacy and Pharmacology, School of Therapeutic Sciences, Faculty of Health Sciences, University of the Witwatersrand, 7 York Road, Parktown, Johannesburg 2193, South Africa

## Abstract

The efficient noninvasive treatment of neurodegenerative disorders is often constrained by reduced permeation of therapeutic agents into the central nervous system (CNS). A vast majority of bioactive agents do not readily permeate into the brain tissue due to the existence of the blood-brain barrier (BBB) and the associated P-glycoprotein efflux transporter. The overexpression of the MDR1 P-glycoprotein has been related to the occurrence of multidrug resistance in CNS diseases. Various research outputs have focused on overcoming the P-glycoprotein drug efflux transporter, which mainly involve its inhibition or bypassing mechanisms. Studies into neurodegenerative disorders have shown that the P-glycoprotein efflux transporter plays a vital role in the progression of schizophrenia, with a noted increase in P-glycoprotein function among schizophrenic patients, thereby reducing therapeutic outcomes. In this review, we address the hypothesis that methods employed in overcoming P-glycoprotein in cancer and other disease states at the level of the BBB and intestine may be applied to schizophrenia drug delivery system design to improve clinical efficiency of drug therapies. In addition, the current review explores polymers and drug delivery systems capable of P-gp inhibition and modulation.

## 1. Introduction

The effectiveness of drug treatments for numerous disease states such as cancer, infectious diseases, and central nervous system (CNS) disorders (epilepsy, depression, and schizophrenia) is limited by poor therapeutic outcomes or drug resistance. The outcomes of drug treatment can be viewed as an interchange of several gene products that have an effect on pharmacokinetics and pharmacodynamics. These gene products mainly include metabolizing enzymes and drug transporters and alterations within any of these gene products may lead to a reduction in clinical outcomes [1]. In particular, the clinical treatment and management of CNS disorders necessitate that a sufficient amount of drug must enter the brain. The use of oral drug delivery systems is beneficial in the treatment of neurodegenerative disorders as compliance to therapy becomes challenging [2]. However, an imperative factor determining the entry of drug molecules into the brain via oral administration is its absorption through the intestinal epithelium and the permeability of the blood-brain barrier (BBB). Passive diffusion across the intestinal epithelium is dependent on many physiochemical characteristics of drugs such as lipophilicity, molecular weight, and hydrophobic bonding [3]. The same principle applies to passive diffusion across the BBB, although passive diffusion across the BBB is limited to small lipophilic molecules. Active efflux of the drug into the intestine and from BBB endothelium back into the blood are the most important mechanisms underlying reduced brain uptake of active drug molecules post oral dosing [4].

The development of drug delivery systems involved in the treatment of neurodegenerative disorders requires a vital consideration of achievable brain concentrations. Factors that impact the brain uptake and concentrations of drugs include (i) the extent of intestinal absorption after oral administration, (ii) the rate and extent of transport across the BBB into the brain, (iii) metabolic stability of the drug, and (iv) the active transport out of the intestine and brain endothelium via efflux pump transporters. There are three classes of transporters that have been associated with the efflux of drugs-monocarboxylic acid transporters, organic ion transporters, and multidrug resistance transporters. This remarkable system of transporters provides a viable mechanism through which the permeation of CNS targeted drugs into the brain is effectually decreased. The action of these efflux transporters at the level of the intestine and BBB may be demonstrated clinically as the reduced effectiveness of drug therapy targeted at CNS disorders [5].

In addition, various multispecific transport proteins have also been identified within the intestine and BBB. Some of these belong to the ATP-binding cassette (ABC) superfamily of transporters with P-glycoprotein (P-gp), multidrug resistance associated protein (MRP), and breast cancer resistance protein (BCRP) as representative examples [4, 6]. P-gp is a membrane transporter of the ABC superfamily located within both the intestinal epithelium and the BBB thus playing a dynamic role in the bioavailability of orally administered drugs employed in the treatment of neurodegenerative disorders [7]. We propose that drug molecules intended for the treatment of CNS disorders must be capable of bypassing the P-gp efflux pump at the intestinal and BBB levels so as to attain effective brain concentrations.

Regardless of the vast advances in brain research outputs, neurodegenerative and psychiatric disorders remain the world's leading causes of disability, morbidity, and mortality [8]. Dysfunctions in the P-gp efflux transporter have already been suggested to play a role in the development of neurodegenerative disorders, such as Parkinson's and Alzheimer's diseases. Genetic variations in the MDR1 gene associated with reduced P-gp function at the BBB have been related to a higher risk of Parkinson's disease. The reduced function of the P-gp efflux pump has been noted in most neurodegenerative disorders. It has been hypothesized that the decreased P-gp function in the BBB may increase the risk and hence the incidence of neurological diseases [9]. In schizophrenia genetic variations of the ABCB1 (ATP-binding cassette subfamily B) gene also known as the MDR1 (multidrug resistance) gene have been described as the predisposing factors for schizophrenia and other neurodegenerative diseases. They have also been employed as determinants of treatment response to antipsychotics [10]. As in other neurodegenerative diseases the BBB maybe compromised by way of the inflammatory and neurodegenerative processes [11]; hence the functionality of P-gp is influenced by the inflammatory responses [12]. As discussed previously, there exists a reduction of P-gp function in progression of neurodegenerative diseases. Conversely, there is evidence of increased P-gp function in patients presenting with schizophrenia [13].

Schizophrenia is a neurodegenerative disorder that is known to affect approximately 1% of the world's population [14], with a further 25% of patients approximated to be afflicted by the disease experiencing nonremitting sickness [15]. The disorder is characterized by disturbances of perception, thought, and volition, with significant impairment in social and occupational functioning. Studies have shown that roughly 10% of schizophrenic patients commit suicide. A recent study showed that more than 70% of patients diagnosed with chronic schizophrenia discontinued their antipsychotic drug therapy, due to poor effectiveness or tolerability [16–22].

**Figure 1:**
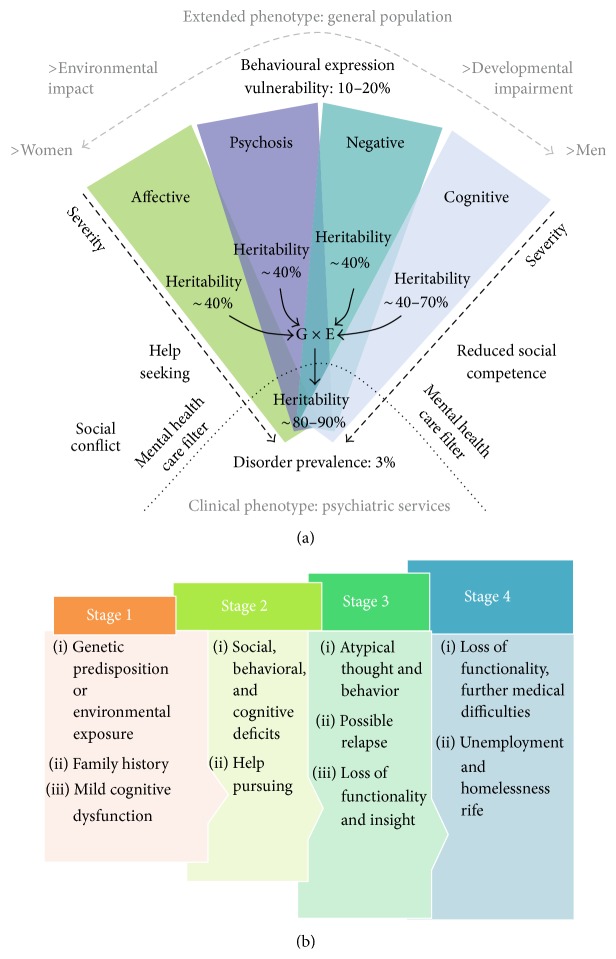
(a) Description of the aetiological complexity of schizophrenia [14] (reproduced with permission from Macmillan Publishers Ltd. Nature 2010). (b) Stages of schizophrenia progression [23] (reproduced with permission from Elsevier B.V. Ltd. © 2009).

Schizophrenia is clinically characterized by the incidence of symptoms such as hallucinations and delusions which are collectively termed “positive” symptoms whereas symptoms pertaining to expression of emotional dullness and social withdrawal are recognized as “negative” symptoms [15].  Figure 1(a) diagrammatically depicts the symptom cascade.

Schizophrenia is widely characterized via a successive course that involves three distinct phases, namely, phase 1 (premorbid) defined by cognitive, motor, and societal dysfunction, phase 2 (prodromal) which is characterized by weakened positive symptoms and accompanying deteriorating functionality, and phase 3 (plateau) in which the positive psychosis defining symptoms are less conspicuous and the negative symptoms including cognitive deficits are generally predominant [23].  Figure 1(b) illustrates the clinical stages of schizophrenia progression.

It has been discovered that “pharmacoresistant schizophrenia” is a noteworthy obstacle to the successful management of the disease within the clinical setting. The prevalence of pharmacoresistant schizophrenia is understood to be approximately 12.9 to 48% of all schizophrenic patients and persists regardless of the use of drug combination treatment regimens with possible therapeutic efficacy. Majority of patients presenting with treatment resistant schizophrenia are on atypical antipsychotic medication. Recent* in vitro* studies have shown that drugs such as amisulpride, risperidone, quetiapine, and clozapine have a varying degree of affinity for the P-gp efflux transporter [24–26]. An additional study was conducted to determine the effect that cyclosporine A, a P-gp inhibitor, has on the brain uptake of amisulpride (a recognized P-gp substrate).* In vitro* and* in vivo* results displayed that amisulpride when coadministered with 50 mg/kg of cyclosporine A showed an increase and prolonged antipsychotic effect. The area under the curve (AUC) in serum and the brain was increased in cyclosporine cotreated rats. These data points indicated a pharmacokinetic interaction between cyclosporine A and amisulpride which is most likely due to P-gp inhibition [27]. Thus, the expression and function of the P-gp efflux transporter have been recently implicated in the occurrence of pharmacoresistant schizophrenia. Antipsychotic drugs have also been shown to stimulate the catalytic activity of P-gp providing additional evidence of the participation of P-gp-mediated drug extrusion processes in restraining CNS penetration of these drugs. It has been suggested that some of the principles observed with the combination treatment of P-gp-mediated drug resistance in cancer may be applied to elucidate the involvement of P-gp in drug-resistant schizophrenia. There exists a greatly emergent body of evidence supporting the role of P-gp on the efflux of antipsychotic agents from the BBB; however limited consideration has been focused on the modulation of P-gp function by polymeric materials, excipients, and drug delivery systems used in other P-gp modulated treatment-resistant disease states [24].

This review will provide a brief overview of the efflux transporters associated with the intestine and BBB, followed by P-gp efflux transporter's mechanism of action, its role in CNS drug delivery, and current and potential schizophrenia therapy. Additionally, the review will provide a discussion on pharmaceutical excipients and novel drug delivery systems employed in other CNS related disease states that have a possible future application in schizophrenia due to P-gp modulation or inhibition.

## 2. Structure and Function of the Intestine and P-gp Intestinal Expression in Drug Delivery

The absorption of drugs via the oral route is always under examination due to the fact that good bioavailability indicates that the drug is able to reach systemic circulation. Oral drug absorption is affected by both drug properties and the physiology of the gastrointestinal tract (GIT) [28]. Oral bioavailability is a collective outcome of fraction absorbed, fraction escaping gut-wall elimination, and the fraction avoiding hepatic elimination. The factors that affect the bioavailability are divided into physiological, physiochemical, and biopharmaceutical factors [29]. The sequence of actions determining the systemic availability of drug molecules subsequent to their oral administration is well acknowledged. However, for particular drugs, the process leading to drug absorption and bioavailability is complex. Certain mechanisms may involve poor drug solubility, poor permeability, degradation (enzymatic/nonenzymatic), and first pass hepatic metabolism [3]. Following oral dosing, drug molecules cross the luminal membrane via a diversity of mechanisms that comprise of passive diffusion or active transport. Passive diffusion is divided into two pathways, the paracellular and transcellular pathway, in which the paracellular pathway drug molecules are absorbed via diffusion and convection volume flow through the water filled intercellular space [29].

The active transport pathway is mediated by transporters and is divided into influx and efflux. The applicability of the various routes is determined by the compounds physiochemical properties and their relative affinity for various transport proteins. Enterocytes express several transporters belonging to the Adenosine triphosphate (ATP) binding cassette (ABC) superfamily and solute carrier (SC) super families, on the apical and basolateral surface of membranes with the function of influx and efflux of endogenous substances. A wide diversity of transporters are expressed in enterocytes; however, only a select few have been implicated in the intestinal absorption of drugs. ABC transporters utilize ATP to power the transport and are thus called primary active transporters. Solute carrier (SLC) transporters employ ion gradients (H^+^, Na^+^, and Ca^++^) across the membrane by active carriers. ABC transporters that are expressed in the intestine include P-glycoprotein (P-gp), breast cancer resistant protein (BCRP), and multidrug resistance protein (MRP), which are localized on the brush border membrane of enterocytes. Efflux transporters mechanistically limit the enterocytic levels of their respective substrates by reducing uptake and facilitating efflux, whereas SLC transporters which include peptide transporter (PepTI) and organic anion polypeptide transporter (OATP1A2) amongst others are secondary active transporters that employ energy coupling mechanisms [29, 30].

**Figure 2:**
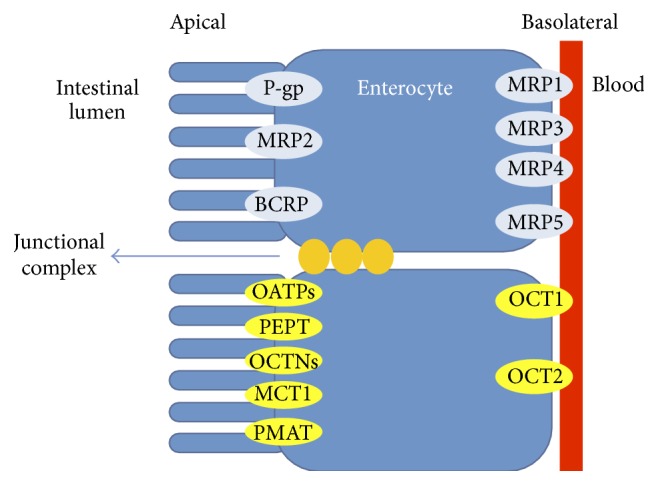
Drug Efflux by P-glycoprotein in the intestine [115] (reproduced with permission from Elsevier B.V. Ltd. © 2013).

During oral absorption, in addition to the physical processes mentioned above (solubility, permeability, etc.), P-gp-mediated efflux across the apical membrane has an effect on the rate and concentration of drug diffusing across the basolateral membrane and thus entering general circulation from the intestine. It has been estimated that 50–60% of new and 40–50% of existing drugs molecules are lipophilic in nature and more than 25% of these agents are known P-gp substrates [2]. Pumps in the intestinal epithelium transport substrates from the intestinal lumen to the general blood circulation [31]. P-gp identifies with a diversity of structurally and pharmacologically dissimilar hydrophobic substances. The P-gp efflux pump transporter restricts the influx into and facilitates efflux from enterocytes of its substrates. Therefore, the P-gp efflux transporter is recognized as a determining factor of oral bioavailability and intestinal efflux of drugs [3]. In the human intestine, P-gp is highly expressed on the apical surface of superficial columnar epithelial cells of the colon and ileum (Figure 2) while expression decreases proximally into the jejunum, duodenum, and stomach [3]. Since the oral route of drug administration is the most preferred as previously mentioned, it is vital to overcome the absorption barrier posed by the P-gp efflux transporter [2]. Additionally, as discussed earlier P-gp plays a role in restricting cellular uptake of drugs from the general blood circulation into the brain due to its presence within the BBB as well. Research outputs have shown that intestinal P-gp can be inhibited by various compounds resulting to an increase in oral absorption of P-gp substrate drugs [3].

Physiological factors that influence oral drug absorption are absorption rate of a drug, which is a function of drug absorption via the GIT and gastro intestinal transit time, which influences the systemic exposure of rapidly, dissolved, and highly absorbed drugs. Intestinal transit time impacts the absorption of drugs with limited mucosal permeability and carrier-mediated uptake and drugs that are subjected to intestinal degradation. In addition, the degree of ionization plays a key role in determining drug dissolution rate and the passive permeability across the GIT. The pH at the absorption site is also a vital factor in allowing or inhibiting the dissolution and absorption of many ionizable drugs [29].

In addition, there are biopharmaceutical factors that influence oral drug absorption; amongst these is the salt form of a drug molecule which changes the coulombic attraction between the drug molecule and its respective counter ion thus altering the potential energy when in a solid state. This is usually associated with an alteration of the pH of the diffusion layer at the surface of the dissolving solid, thereby greatly increasing the solubility of the drug molecule. The amorphous form alternatively tends to improve the dissolution rate and solubility significantly compared to its crystalline form which increases the rate and degree of oral absorption [29]. Apart from these mechanisms, the small intestine has the ability to metabolize drugs via a diversity of pathways involving phase I and phase II reactions, which in turn may cause a restriction in oral bioavailability. CYP3A4 is the most abundant cytochrome P450 enzyme within the intestinal enterocytes that is implicated in the metabolic elimination of many drugs [29].

## 3. Structure and Function of the BBB and P-gp Expression in Drug Delivery

**Figure 3:**
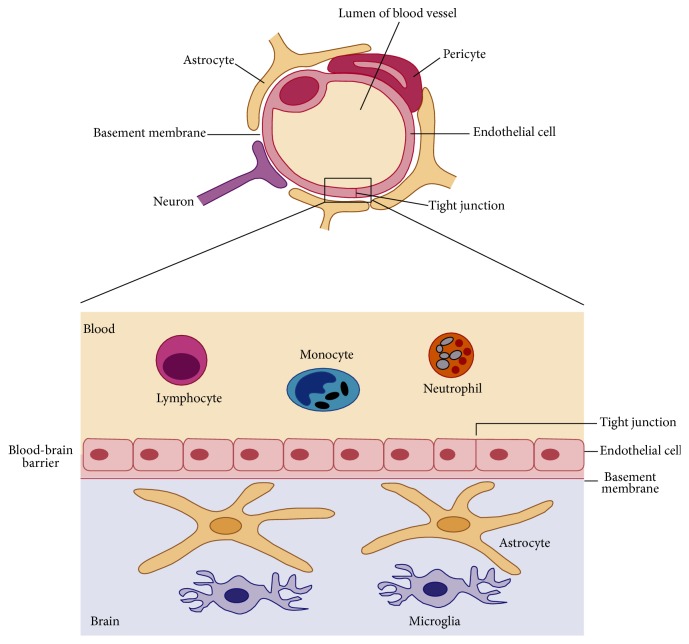
Schematic representation of the cross section of the BBB cerebral capillary [116] (reproduced with permission from Elsevier B.V. Ltd. © 2007).

The BBB is a specialized system of capillary endothelial cells as shown in Figure 3, which assists in protecting the brain from harmful substances within the blood stream, while supplying the brain with nutrients that are necessary for proper function. The BBB has many functions, including maintenance of the internal environment of the brain, protection of the brain from fluctuations in ionic composition, and drainage of cerebrospinal fluid and interstitial fluid. Due to its protective function the BBB restricts transport of substances into the brain via both the physical (tight junctions) and the metabolic (enzyme) barriers [32, 33].

**Figure 4:**
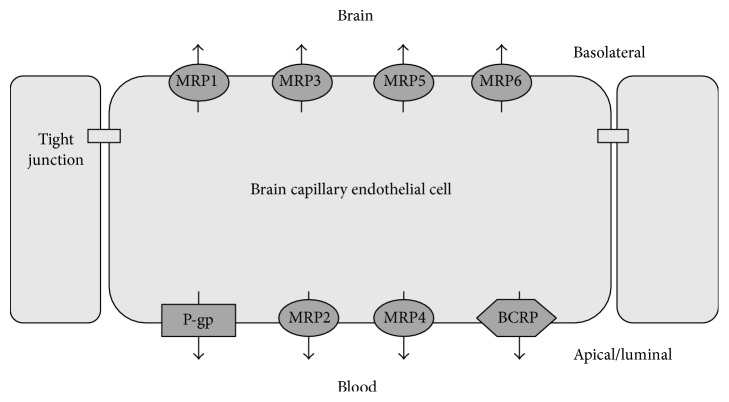
Respective locations of the drug efflux proteins on brain capillary endothelial cells that collectively form the BBB [35] (reproduced with permission from Elsevier B.V. Ltd. © 2005).

The BBB is composed of a monolayer of brain capillary endothelial cells. The limitation of drug uptake by the BBB arises via the presence of tight junctions between adjacent capillary endothelial cells and the relative lack of fenestrae as well as pinocytotic vesicles within the endothelial cell monolayer. In turn, brain capillary endothelial cells are surrounded by an extracellular matrix, pericytes, and astrocyte foot processes. Once a drug molecule enters the brain, either via transport across the BBB after systemic administration or by direct administration into the CNS, the drug may return to the blood via three ways: (1) It may pass through the capillary endothelial cells of the brain, (2) cross the epithelial cells of the choroid plexus, (3) or return to systemic circulation by bulk flow of cerebrospinal fluid that effectively results in reabsorption at the arachnoid villi [5]. The BBB is known to express a high degree of active efflux transporter proteins, including, as mentioned previously, P-gp, MRP-1, and BCRP. Lipophilic molecules of approximately 500–600 Daltons may diffuse passively into the CNS. However, the vast majority of molecules of a molecular weight greater than 600 Daltons do not cross the BBB (Figure 4). Therefore in order to reach the brain, most drug molecules must cross the BBB via an interaction with specific transporters occurring at the luminal surface of the endothelial cells [1, 34–36].

The pharmacodynamics and pharmacokinetics of CNS targeted drugs are determined by their unbound concentrations in the extracellular fluid of the brain. Several* in vivo* and* in vitro* procedures are accessible to study these properties. The efflux transport across the BBB is an imperative process utilized in the explanation of the mechanism of the apparent restricted cerebral distribution of drugs after their systemic administration. To examine the BBB efflux transport mechanism under* in vivo* conditions, an intracerebral microinjection technique has been developed and newly estimated as the BEI (Brain Efflux Index). BEI is defined as the relative percentage of drug that is effluxed from the ipsilateral (does not cross the opposite hemisphere of the brain) cerebrum to the circulating blood as compared with the amount of drug injected into the cerebrum as shown:

(1) $$\begin{array}{c} \text{BEI}\%=\frac{\text{Amount of drug effluxed at the BBB}}{\text{Amount of drug injected into the BBB}}\times 100. \end{array}$$

However, limitations of the BEI method are that only one data point may be obtained for a single intracerebral injection, and the concentration the drug in the cerebrum cannot be accurately determined [37].

The net uptake of a drug molecule by the brain via the BBB depends on the overall difference between the uptake and efflux processes (Table 1). Brain uptake of drug molecules is controlled by various factors, including the systemic disposition of the drug as well as the properties of the endothelial cells. Thus, permeability of endothelial cells and their ability to metabolize drugs actively regulate the amount of drug crossing the BBB in both directions [38].

In recent years it has become well perceived that the activity of the P-gp in the BBB plays a restrictive role with regard to the net cerebral uptake of many therapeutic drugs. Owing to its unspecific substrate affinities, P-gp restricts the achievable cerebral concentration of various medications and may be a crucial factor in the phenomenon of pharmacoresistance [39–41]. P-gp is a prototypical multidrug resistance (MDR) transporter; its discovery was based on its ability to confer drug resistance to cancer cells. P-gp, a phosphoglycoprotein, acts as an unrestrained energy-dependent efflux pump [5]. Given that P-gp efflux accountability can be a key obstacle for CNS therapeutic drugs to cross the BBB and reach the target, the interactions of the CNS targeted drugs with the P-gp transporter may lead to the lack of CNS activity as a result of the minimized brain penetration. Therefore the estimation and understanding of the significance of P-gp-mediated efflux transporter have become an important stage in the discovery and development of CNS delivery strategies [42].

## 4. P-gp Efflux Transporter Structure and Mechanism of Action

**Figure 5:**
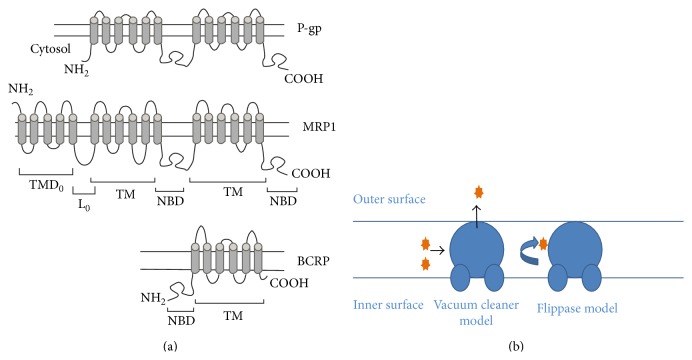
(a) Structural configuration of P-gp, MRP, and BCRP [47] (reproduced with permission from Elsevier B.V. Ltd. © 2005). (b) Diagrammatic representations of the “Vacuum Cleaner” and Flippase model of P-gp function [48] (reproduced with permission from Frontiers in Oncology Ltd. 2014).

In response to the inefficiency in conventional delivery mechanisms, a great amount of research efforts have lately focused primarily on the development of new strategies to allow for a greater efficacy in the delivery of drug molecules to the CNS. In particular, these research efforts have focused on the modulation of P-gp efflux transporters within the BBB [43]. P-gp is presently the most widely studied member of the ABC transporter family and has been shown to transport an extensive list of substrates with a great degree of structural diversity [44]. P-gp is a type of energy-dependent ATPase transmembrane drug efflux transporter. It is a 1280-long amino acid glycoprotein, expressed as a single chain structure containing two homologous portions of equal length, each portion consisting of 6 transmembrane domains and two ATP binding regions separated by a flexible polypeptide linker portion [45, 46], (Figure 5(a)), [47, 48].

There are numerous models to explain the mechanism of efflux by the P-gp transporter. The three most predominant models, pore, flippase l, and the hydrophobic vacuum cleaner model, are commonly used to explain the mechanism of efflux (Figure 5(b)). The vacuum cleaner model hypothesizes that P-gp recognizes substrates embedded in the inner leaflet of the plasma membrane; the substrate is then transported through the protein channel out of the cell [48]. Within the perspective of this model, P-gp binds directly to the substrate molecules that are located on the plasma membrane and pumps them out of the cell by recognizing the substrates as foreign bodies [49]. The pore model is described as an interaction of P-gp with drugs within the boundaries of the cytosol, followed by efflux out of the cell via protein channels. The hypothesized flippase model explains drug efflux as a “flipping” of drug molecules from the inner leaflet to the outer leaflet of the cell against a concentration gradient. These drug molecules then undergo diffusion to reach the extracellular space [49]. However, the flippase model is the most generally received model and increasing amounts of evidence have made it the most favored amongst all [50–52].

## 5. P-gp in Schizophrenia Therapy

Antipsychotic drugs and other pharmacological agents used to treat psychiatric illness must cross the BBB to reach the target sites within the CNS to be able to exert their therapeutic effects. Antipsychotic drugs are crucial treatment options for symptoms of schizophrenia. The first generation or conventional antipsychotics include drugs that have high affinity antagonism of dopamine D_2_ receptors. The second generation antipsychotics or atypical agents include drugs with a lower D_2_ receptor affinity and a far greater affinity for other neuroreceptors [53]. Recently, it has become progressively evident that drug transporters situated within the BBB play a fundamental role in the pharmacokinetics of several drugs with therapeutic implications.

There are strong indications for an impaired integrity of the BBB in schizophrenia, such as increased intracerebral concentrations of the soluble intercellular adhesion molecule-1 (sICAM). Furthermore, the concentration of immunoglobulin and albumin is raised in cerebrospinal fluid in 30% of schizophrenic patients. P-gp function has been reported to be reduced in the progression of neurodegenerative diseases [54].

Wang and coworkers [55] conducted a study employing the abcb1a/b knockout mouse model with a functional deficiency in P-gp. By utilizing ATPase activity as a marker for P-gp activity, an* in vitro* study provided evidence that various atypical antipsychotic drugs such as risperidone and olanzapine may be effectively effluxed by P-gp. In a subsequent* in vivo* study utilizing the abcb1a gene knockout mouse model, results depicted that P-gp restricted the brain penetration of olanzapine, as the resulting brain concentration was 3-fold higher in the abcb1a knockout mice than as the comparative control mice [55].

Risperidone is an atypical antipsychotic drug that has potent antagonistic affinities toward serotonin 5-HT_2_ and dopamine D_2_ receptors. Risperidone is recognized for its effectiveness against both positive and negative symptoms of schizophrenia. An* in vitro* study by Nakagami and coworkers [56] of the activity of P-gp against a range of antipsychotic agents showed that P-gp is likely to influence the absorption of all atypical antipsychotics to various degrees [56].

In addition to other studies relating to P-gp function the activity of the P-gp transporter at the level of the BBB can be studied* in vivo* via Positron emission tomography (PET) with [^11^C] verapamil as a PET tracer. PET seems to be a suitable means of* in vivo* valuation of P-gp functionality in humans and allows for the quantification of BBB/P-gp function in neurodegenerative disorders. The PET brain imaging study with the use of [^11^C] verapamil as a radiotracer was conducted on schizophrenic patients and a comparative healthy control group. Data obtained from the study showed a regionally reduced [^11^C] verapamil uptake within the temporal cortex and basal ganglia of schizophrenic patients versus healthy controls. These results have been interpreted as an increase in P-gp function in the brain of schizophrenic patients. These findings are of immense worth since an increase in P-gp function may be pertinent for the clinical progression of schizophrenia. An increase in P-gp function leads to reduced uptake of antipsychotic agents and may thus be associated with poor clinical outcomes [54].

A further study by Kirschbaum and coworkers [57] found that the ATPase activity assay displayed high affinity of the antipsychotic risperidone for P-gp.* In vivo* studies conducted in mice and humans revealed variations in brain and serum concentrations for both risperidone and its active metabolite, 9-hydroxyrisperidone, which was recently approved as a new antipsychotic paliperidone. In comparison, the conventional antipsychotic haloperidol which displays a high antagonistic activity at dopamine D_2_ receptors is poor substrate of P-gp. Behavioral changes in the mdr 1a/1b knockout mice in comparison to the wild-type mice allow for the investigation of P-gp-dependent variations in the expression degrees of the CNS which leads to functional dispositions. Motor impairment on a rotarod physical task is characteristic of antipsychotic related D_2_ receptor antagonism [54, 57].

This very hypothesis was applied to mdr 1a/1b and wild-type mice after administration of either the known substrate risperidone or a poor substrate haloperidol. Results revealed distinctively different profiles in motor effects by the antipsychotic drug risperidone which was dependent on P-gp expression. Brain concentrations of risperidone and its 9-hydroxyrisperidone metabolite were 10-fold higher for the first three hours in knockout mice versus wild-type mice. The increased concentration reflects the change in dose response seen in the behavioral task. The results establish for the first time the strong association between physical functionality and P-gp-dependent brain concentrations. Pharmacokinetic data of this study displayed a 10-fold higher brain level of risperidone and a 20-fold higher brain level of 9-hydroxyrisperidone in mdr1a/1b mice as compared to wild-type mice. Conclusively, antipsychotic drugs are effectively transported by the P-gp efflux transporter [57].

In conclusion, an additional study was conducted on the atypical antipsychotic aripiprazole. To assess the role of P-gp in the distribution and penetration of aripiprazole within the BBB, mice deficient in the P-gp gene were dosed intraperitoneally with 2 *μ*g/g of aripiprazole. Wild type mice were administered the same dose as the gene deficient mice. The results showed that deficiency of P-gp had a drastic effect on drug concentrations within the brain. Thus aripiprazole has been characterized as a transportable substrate of P-gp [53].

## 6. P-gp Substrates and Inhibitors

In an attempt to estimate the effects of P-gp* in vivo*, a range of* in vitro* P-gp assays have been developed so as to categorize compounds as P-gp substrates or inhibitors (Table 2). One such assay is the Transwell-based assays using polarized cell lines such as Madin-Darby Canine kidney (MDCK) cell line. The MDCK cell line may be stably transfected with human MDR1 or Mdr1a. Comparing the efflux ratios between MDR1-MDCK and MDCK, Transwell assays are able to provide a measure of the specific human P-gp-mediated efflux activity [57].

Another generally used P-gp* in vitro* assay is the P-gp ATPase assay for assessing drugs that demonstrate P-gp interactions as substrates. The standard reagent of the ATPase assay is a membrane preparation from insect cells that are vastly expressive of human P-gp. Functional human P-gp will transport P-gp substrates across the membrane thus resulting in the release of inorganic phosphates [58]. In addition, the* in vitro* calcein AM P-gp inhibition assay can be utilized to detect compounds that inhibit the P-gp-mediated efflux, via employing a fluorescent P-gp substrate, calcein. The calcein assay is capable of differentiating between various P-gp inhibitors from noninhibitors by measuring the resultant fluorescence of calcein [58].

A further* in vitro* approach in studying the P-gp activity is the use of P-gp knockout mice in comparison to wild-type mice. P-gp knockout mice are genetically modified animals in which both genes that are homologous to the human MDR1 gene have been disrupted, resulting in a viable line of animal models. An assessment of the brain plasma area under the curve (AUC) ratio in knockout mice as compared to wild-type mice has become a standard experimental methodology to determine whether P-gp-mediated efflux poses a possible obstacle to the activity of CNS targeted drugs* in vivo* [1, 59, 60].

P-gp exhibits significant capability to bind and transport a wide range of structurally and functionally dissimilar compounds. Typical drug substrates include typical and atypical antipsychotics as well as anticancer agents. P-gp also interacts with yet another group of hydrophobic agents, known as chemomodulators, that is, calcium channel blockers and phenothiazine's. Chemomodulators belong to two categories, those that are transport substrates themselves and thereby compete for efflux and those that exert their effects by binding to and blocking drug efflux without themselves being transported out of the cell [24].

In general, P-gp can be inhibited via (i) blocking the drug binding site competitively/noncompetitively/allosterically, (ii) by interfering with ATP hydrolysis, and (iii) by altering the integrity of the cell membrane lipids [45]. Based on their specificity and affinity, P-gp inhibitors are categorized into three generations: First generation inhibitors are pharmacological actives, which are in clinical use for other indications but have been shown to inhibit P-gp. Second and third generation inhibitors specifically modulate P-gp, with the second generation inhibitors lacking the pharmacological activity of its first generation counterparts and with a higher affinity for P-gp. The third generation inhibitors have a 10-fold higher affinity and potency to inhibit P-gp [24].

## 7. Novel Drug Delivery Systems and Pharmaceutical Excipients Capable of CNS and Intestinal Delivery via P-gp Bypass or Inhibition with a Potential Applicability in Schizophrenia Therapy

### 7.1. Formulation Excipients Utilized as P-gp Inhibitors

Approaches to bypass the P-gp efflux pump may be to combine the target drug with either another P-gp pump substrate or an inhibitor; on the other hand there is the novel approach of combining the active drug molecule with a lipid or polymer excipient which is capable of P-gp inhibition. The initial approach is effective in its inhibitory potential; however it involves the use of a second pharmaceutically active agent that increases the risk of drug-drug interactions; thus in retrospect the use of inhibitory excipients has nonspecific pharmacological action and therefore the possibility of systemic effects and drug interactions is eliminated [50].

### 7.2. Chemosensitizers

Chemosensitizers, also termed reversal agents, are one such approach; they inhibit the efflux pump and increase the absorption of drugs intracellularly; and thus the coadministration with clinically relevant compounds has become a common practice [61]. Chemosensitizers function by acting as competition at the P-gp substrate binding site or by blockade of ATP hydrolysis; drugs such as verapamil, cyclosporine A, and antisteroidal agents such as tamoxifen are classified as 1st generation P-gp modulators. However high concentrations of these agents are required for this purpose and thus lead to pronounced side effects; thus the development of 2nd generation chemosensitizers was prompted. This nevertheless proved to exhibit the same limitations.

To circumvent limitations observed with the 1st and 2nd generation P-gp chemosensitizers, 3rd generation chemosensitizers have been developed utilizing the supremacy of combinational chemistry and Structure Activity Relationships (SARs). These agents are noncompetitive inhibitors that result in alterations in protein conformation, thus modulating P-gp substrates; they include elacridar and tariquidar amongst others [62]. Further research conducted depicted evidence that drug delivery systems in combination with P-gp modulators could considerably enhance pharmacokinetics of the P-gp modulator and in addition reduce the pharmacokinetic interactions with P-gp substrates. Cremophor EL was one of the excipients employed in the above mentioned study which showed promising activity as a P-gp modulator [62]. Furthermore, in an experimental study conducted by Choo and coworkers tariquidar, a P-gp modulator in combination with propylene glycol, 5% dextrose, and ethanol, was able to inhibit P-gp function at the BBB in high doses [63], thus displaying a possible application in improving CNS permeability of drugs employed in neurodegenerative diseases. In another research study it has been shown that GF120918 (elacridar) a known 3rd generation P-gp modulator is capable of increasing the brain uptake of paclitaxel (P-gp substrate) 5-fold [32].

### 7.3. Natural Polymers

The natural gums are classified as polysaccharides that consist of multiple sugar groups that bonded together to create much larger molecules; they offer the advantages of being sustainable, biodegradable, and biologically safe; these natural gums are known to form gels that depict a high degree of network formation. Gums are capable of undergoing grafting and crosslinking reactions and through such reactions they may form hydrogels, xerogels, and nanoparticles which have a very important function in the inhibition of the P-gp efflux transporter [63].

Chitosan, a cationic polysaccharide in combination with nucleotides to form nanosystems, may enhance the permeability of drug molecules into the CNS via opening of tight junctions, and the formulation of nanosystems incorporating chitosan and nucleotides will enable this purpose [64].

Natural polymers such anionic gums, for example, xanthan and gellan gum, as well as alginates, have been shown to inhibit the action of the P-gp efflux pump at concentrations of 0.05% and 0.5 mg/mL, respectively [65]. In the presence of xanthan gum studies have shown increased concentration of P-gp substrates such as vinblastine and doxorubicin. Alginate flavicam demonstrated an increase in the intracellular concentration of doxorubicin in everted gut sac cells [66].

### 7.4. Synthetic Polymers

Synthetic polymers are synthesized via polymerization reactions and the modification of natural polymers or via the complexation of natural polymers with naturally occurring agents such as fatty acids [66]. Synthetic polymers such as PEG 400, at concentrations between 1 and 20%, increased accumulation of digoxin due to the inhibition of the P-gp drug efflux pump [67, 68]. Similar studies conducted employing Pluronic P85 and Vitamin E D-*α*-Tocopheryl polyethylene glycol 1000 succinate on the digoxin uptake in rats have further proven that pluronic blocked copolymers are capable of inhibiting the P-gp efflux pump and enhancing substrate uptake [69]. Furthermore, studies have shown that pluronic P85 has an inhibitory action on the ATPase enzyme that is involved in the functioning of the P-gp efflux pump proteins, thus resulting in inhibitory reactions between these proteins and the P-gp substrates [70]. In a study conducted by Batrakova and coworkers who have shown the increased brain concentration of digoxin in mdr1 knockout mice and wild type mice treated with Pluronic P85, it was clearly demonstrated that Pluronic P85 delayed the residence time and concentration of digoxin in the brain [71]. Commonly occurring polymers that act as P-gp inhibitors are listed in Table 3.

Research studies have been conducted to evaluate the inhibitory properties of PEG on the P-gp efflux pump. The study involved the grafting PEG to polyethyleneimine (PEI) and then undergoing a thiolation reaction employing *γ*-thiobutyrolatone to formulate a novel grafted thiomer. The grafted PEG was then evaluated based on its effect on the transport of Rhodamine–123, a well-known P-gp substrate. Results depicted that the cellular uptake of Rhodamine-123 was improved with the grafted-thiolated PEG as compared to free drug solutions.

Oral administration of many drug substances is limited due to the poor bioavailability and systemic uptake which is directly linked to the presence and action of the P-gp efflux pump. In an attempt to overcome this limitation there has been the development of P-gp pump inhibitors; however due to the abovementioned set back of requiring high doses, none of them have been approved for oral administration due to the risk of systemic side effects. Thus polymeric P-gp inhibitor incorporation into drug delivery systems as excipients provides a valuable approach; they show a high inhibitory potential and due to their great molecular weights they are not absorbed from the gastrointestinal system and thus eliminate any possible systemic effects. Previous studies determined the inhibition of P-gp by PEG's by their effect on the elevated permeability of substrate drug molecules such as taxol and doxorubicin within caco-2 monolayers [72]. PEGylated sorbitans that are esterified with fatty acids are commonly known as polysorbates. The most extensively studied group of the Polysorbates is polyoxyethylene sorbitans monolaurates, known by the trade name Tween; these agents have been found to enhance the accumulation of daunorubicin within tumor cells [66].

Poloxamers also fall within the category of synthetic polymers and are also known as Pluronics. Specifically, Pluronic P85 has been studied for its inhibitory effect on the function of the P-gp efflux pump. Two main areas that P85 is used for its inhibitory properties are in the BBB and cancer chemotherapy [66]. The mechanism of inhibition has been dedicated to a reduction in ATP and inhibition of ATPase enzymes as well as lipid membrane fluidization [70, 71]. Pluronic brings about a reduction in ATP levels within bovine endothelial cells; the ATP depletion was reasoned to be as the result of the inhibition of cellular metabolism [73]. Additionally, mixed micelles synthesized from a combination of poloxamer 407 and D-*α*-Tocopheryl polyethylene glycol 1000 succinate (TPGS 1000) have the potential of elevating the clinical efficiency of drugs by increasing the amount of drug in the interior of the cell by inhibiting P-gp efflux transporters [74]. TPGS 1000 is classified as a water soluble vitamin that consists of a lipophilic head and hydrophilic PEG tail. The mechanism by which TPGS 1000 inhibits the P-gp pump has been proposed as a combination of membrane fluidization, ATP depletion, and prevention of substrate binding. Research on the effect of pluronic block polymers on the accumulation of digoxin has proven the hypothesized inhibition of P-gp efflux transporters by Pluronic polymers. The treatment of various substrates with Pluronic polymers displayed an increase in apical to basolateral transport, thus displaying P-gp inhibition [66, 70, 71].

**Figure 6:**
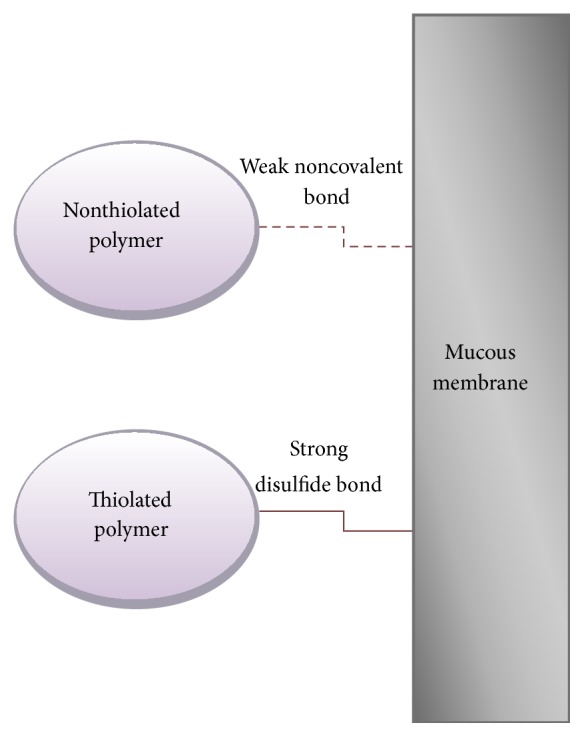
Mechanism of inhibition of the P-gp efflux pump [117] (reproduced with permission from Elsevier B.V Ltd. © 2013).

Thiomers, yet another group of synthetic polymers, are characterized as polymers containing a thiol moiety within their chemical structure; the presence of the thiol moiety allows for the formation of disulfide bonds between the cysteine groups, of the P-gp pump, for example, *α*-chitosan-thiobutylamidine [75]. The mechanism of thiomer mediated inhibition of the P-gp efflux pump is depicted in Figure 6. Improved transmucosal transport of Rhodamine-123 a P-gp substrate in the company of thiolated chitosan, produced by linking of amino groups of chitosan and thiol groups [76], was displayed by Bernkop-Schnurch et al. due to P-gp transporter inhibition. Thiomers are not absorbed from the GIT due to their high molecular weight; therefore systemic side effects are eliminated and their use as pharmaceutical excipients for their P-gp inhibitory properties is greatly beneficial [72]. As described above the P-gp efflux pump is made up of 12 transmembrane units; these units in turn form channels through which P-gp substrates pass through to exit the cell; two of the 12 units display cysteine subunits. Thiomers are theorized to enter the channels of the P-gp pump and thereafter form disulfide bonds with the cysteine subunits; in this manner the transport of drug molecules out of the cell is hindered and thus there is an inhibition of P-gp efflux pump action [64]. Thiolated chitosan is a directly compressible excipient which may therefore be formulated into matrix tablets [76].

### 7.5. Solvents and Surfactants

Both surfactants and solvents act by interaction with the polar heads of the lipid bilayers thereby adjusting hydrogen bonding and ionic forces and have the potential to insert themselves between the nonpolar tails of the lipid bilayer. These interactions cause the fluidization of the lipid membrane and thus P-gp inhibition. Nonionic surfactants such as Tween and Span possess P-gp transporter inhibitory potential; these agents are also hydrophobic and thus rendered less toxic [77]. Surfactants are furthermore important in the formulation process of nanoparticles. Various research outputs have shown that the efficiency of surfactants as P-gp inhibitors is based on their respective chemical structures. Surfactants such as Solutol HS15, Tween 80, and Cremaphore EL contain Polyethylene glycol on the hydrophilic portions of their structures, which have displayed the ability to increase intracellular concentrations of epirubicin in human colorectal carcinoma cells, thereby confirming that surfactants act as P-gp modulators [62]. In a study conducted by Rege and coworkers [78] the effect of Tween 80, Cremophor EL, and vitamin E TPGS on the P-gp efflux transporter in caco-2 cell monolayers was investigated. Results showed that all three surfactants inhibited P-gp and Tween 80 and Cremophor EL increased the apical to basolateral permeability of Rhodamine 123 (P-gp substrate) within a concentration range of 0-1 mM. Whereas vitamin E TPGS contributed an inhibitory effect at a concentration of 0.025 mM [78].

## 8. Drug Delivery Systems with P-gp Modulatory and Enhanced Intestinal Absorption Applications

The following section will focus on the strategic design of drug of delivery systems studied for their aptitude to enhance bioactive delivery through the intestinal epithelia by P-gp modulation and/or inhibition with a future applicability in schizophrenia therapy.

### 8.1. Bestatin-Improved Intestinal Absorption by Coadministration of a P-gp Inhibitor

A study by Huo and coworkers [79] has shown the enhancement effect of P-gp inhibitors on the intestinal absorption of bestatin. The study showed that when bestatin and cyclosporine A were coadministered orally, the resultant plasma concentration of bestatin was considerably increased. Cyclosporine A and Verapamil, commonly utilized P-gp inhibitors, can eliminate the efflux pump mediated by P-gp, resulting in improved intestinal absorption of the coadministered P-gp substrate. The study by Huo and coworkers [79] displayed a 90% increase in bestatin intestinal absorption seen in the Cmax and AUC with the addition of cyclosporine A [79].

### 8.2. Enhanced Intestinal Absorption of Ganciclovir by P-gp Inhibition Using Pharmaceutical Excipients

Several studies have shown that P-gp inhibitors such as verapamil and cyclosporine A are capable of enhancing the bioavailability of a variety of drugs. However, such inhibitors are pharmacologically active agents and thus may cause toxic effects. In addition, these agents are absorbed into the bloodstream and can interact with P-gp in other organs that it is expressed in.

On the other hand, pharmaceutical excipients, which are utilized as inert excipients in drug delivery formulations, are being investigated as superior class of P-gp inhibitors. Several studies have shown that these excipients can greatly improve the intestinal absorption of a wide range of drugs by P-gp inhibition.

These excipients are nonabsorbable; thus there will be no unnecessary P-gp inhibition in other organs. A study conducted by Li and coworkers [80] studied the effects of common excipients on the intestinal absorption of ganciclovir, a substrate of P-gp in rats. The* in vitro* transferal from mucosa to serosa as well as the* in situ* transepithelial permeation was evaluated. Selected excipients within a concentration range of 0.1–1% w/v were shown to increase the concentration of ganciclovir transported in an everted gut sac model.

Results depicted a significant increase in permeability of ganciclovir by all excipients employed in the study. The effects of EL-35 and F-68 were dose-dependent but that of PEG 400 and Tween 80 were not. The results observed confirm that the improvement of intestinal absorption of ganciclovir via the use of excipients is due to inhibition of P-gp inhibition mediated drug efflux. The mechanism of F-68 inhibition of P-gp is due to its blocking effect on the binding site of the P-gp efflux transporter, whereas PEG 400, tween-80, and EL-35 are due to alteration in the membrane fluidity of P-gp [80].

### 8.3. Self-Emulsifying Drug Delivery Systems

This is another drug delivery approach that may prove useful in bypassing the P-gp pump due mainly to the presence of surfactants such as Tween 80 that have been identified as P-gp inhibitors [78]. SMEDD's consisting of vitamin E (oil phase) and surfactants have been shown to enhance the solubility and bioavailability of paclitaxel (a P-gp substrate) owing to their P-gp inhibitors activity.

An advantage of using SMEDD's is their capability to incorporate and solubilize high concentrations of P-gp inhibitors. Many studies have been conducted to improve the oral bioavailability of this chemotherapeutic agent so as to increase clinical outcomes; it has been proven that P-gp plays a vital role in the bioavailability of paclitaxel; the study conducted by Woo and coworkers shows that the use of KR-30031 a verapamil analog with P-gp inhibitory effects in combination with paclitaxel improved the bioavailability of the later [81]. Nanocapsules have been used as a drug delivery system to improve the oral bioavailability of the P-gp substrate Tacrolimus, which was incorporated into nanocapsules with the use of polymethacrylate polymers. Results depicted that the bioavailability was between a range of 2.45- and 4.9-fold compared to the commercially available formulation [2].

### 8.4. Double Coated Nanocapsules for Enhanced Intestinal Absorption and Bioavailability of a P-gp Substrate

An experimental study performed by Nassar and coworkers [2] proposes the use of double coated nanocapsules to improve the bioavailability of a P-gp substrate, Tacrolimus. Using caco-2 cells and intestinal rat segment the effect of encapsulation of Tacrolimus on P-gp was evaluated. The double coated nanocapsules prevented its molecular recognition by the P-gp efflux transporter in caco-2 cells. The conclusive results show that the nanocapsule delivery system is a great platform for intestinal absorption of lipophilic drugs which are P-gp substrates. The oral bioavailability of Tacrolimus was 4.9- and 2.45-fold greater when compared to a marketed product [2].

### 8.5. Novel Polymeric P-gp Inhibitor for Enhanced Intestinal Drug Delivery

Many drugs are unable to be administered via the oral route owing to poor absorption into the systemic circulation due to intestinal P-gp efflux pumps. In order to circumvent this obstacle many types of efflux pump inhibitors have been developed. Yet, many of these inhibitors have not been approved for oral use due to their systemic side effects. A noteworthy alternative would be polymeric based efflux pump inhibitors, displaying greatly efficient inhibitory action and no systemic side effects because it is unabsorbed into the intestine due to its high molecular mass.

In a study conducted by Iqbal and coworkers [72] a novel thiolated copolymer, thiolated PEG-g-PEI copolymer, was synthesized. The novel thiolated copolymer showed a significantly greater effect on the intestinal absorption of Rho-123, a P-gp substrate as compared to other P-gp inhibitors. In addition, the thiolated copolymer was able to increase the intestinal uptake of Rho-123 up to 3.26-fold in comparison to a system lacking a P-gp inhibitor. In addition to enhancing the absorption of Rho-123 the novel thiomer was also capable of decreasing the basolateral to apical transport across the rat intestinal mucosa segment. The study has thus provided evidence that the novel thiomer has a great applicability in improving bioavailability of P-gp substrate drugs [72].

### 8.6. Influence of Intestinal P-gp on Absorption of Furosemide a P-gp Substrate

Furosemide is a loop diuretic employed in the clinical treatment of congestive heart failure and hypertension. Pharmacokinetics of furosemide show that it has incomplete absorption from the gastrointestinal tract after oral dosing. P-gp efflux pumps have been implicated as the possible cause for reduced absorption; however no complete evidence exists. The intestinal absorption of furosemide was evaluated in the presence and absence of verapamil utilizing the rat intestinal sac model in an experimental study conducted by Al-Mohizea [82].

Results obtained from the study depict low drug permeability in the noneverted rat intestinal sac. This replicates the drug transference from the mucosal to serosal region which imitates the* in vivo* environment. In contrast, when observed in the everted rat intestinal sac, drug displayed a noticeably greater transport as compared to transport across the noneverted intestinal sac. These results clearly indicate the involvement of the P-gp pump and because the defined function of the P-gp efflux transporter is to pump drug back into the intestine subsequent to absorption, the observed outcomes indicate a greater transintestinal permeation from the serosal to mucosal region. The ratio of serosal to mucosal and mucosal to serosal permeability was discovered to be 5.6 and this was advocated for the possible role of P-gp efflux transporter in the intestinal uptake of furosemide. The effect of verapamil hydrochloride, a P-gp efflux pump inhibitor, on furosemide absorption was employed to confirm the results obtained from the intestinal sac model experiment.

It was shown that the presence of verapamil increased the mucosal to serosal transport of furosemide noticeably compared to the control lacking verapamil. This observation is due to the inhibitory effect of verapamil on the P-gp efflux pump, which prevents transport of drug back into the mucosal region. The ratio of serosal to mucosal and mucosal to serosal permeability was reduced from 5.6 in the absence of verapamil to 1.28 in its presence. These results confirm the mechanistic effect of the P-gp efflux pump on intestinal absorption. Furthermore, the current study evaluated the effect of Tween 80 and HP*β*CD, two commonly employed pharmaceutical excipients, on the intestinal uptake of furosemide. The results displayed a permeation improvement effect for Tween 80 but not for HP*β*CD. This in turn inferred a substantial increase in the mucosal to serosal permeability of furosemide. Tween 80 was shown to decrease the ratio of serosal: mucosal to mucosal: serosal permeability (5.6 to 1.08). This provides evidence that Tween 80 is a noteworthy P-gp efflux pump inhibitor [82].

## 9. Drug Delivery Systems with P-gp Modulation and Enhanced BBB Absorption

The following section will focus on drug delivery systems that have been studied for their unique ability to enhance BBB permeability of drug agents via P-gp inhibition and/or bypass with a prospective application in schizophrenia therapy. The effective noninvasive treatment of neurological diseases is frequently restricted by poor access of bioactives into the central nervous system (CNS). Many therapeutic agents do not freely permeate into brain tissue due to the presence of the BBB and its associated P-gp efflux pump transporter. Recent advances in drug delivery technology have provided promising solutions to overcome these challenges.

The use of drug delivery systems with the ability to bypass or inhibit the P-gp efflux transporter has shown a great improvement in CNS bioavailability and hence enhanced therapeutic outcomes. Furthermore, nanocarriers ranging from polymeric nanoparticles, solid lipid nanoparticles, liposomes, dendrimers, and micelles have been studied for their ability to deliver CNS therapeutics and have displayed success in the treatment of CNS diseases such as brain tumors and alzheimer's [83]. Hence, we propose the use of these delivery systems in the treatment of schizophrenia so as to bypass the BBB and its efflux transporters.

### 9.1. Nanocarriers

Nanoparticles can gain access to the brain via numerous mechanisms. These mechanisms acting either alone or in combination can explain the transport of therapeutically bioactive agents across the BBB: (i) adsorption and pooling of nanoparticles in the capillaries of the brain, followed by adsorption to the capillary wall, which provides a concentration gradient across the blood capillaries located in the brain cells; therefore this may enhance the transport of bioactives across the endothelial cell monolayer and thus increase the uptake of the bioactive into the brain, (ii) receptor-mediated endocytosis of nanoparticles across the BBB, followed by internalization of the complex and release of the bioactives within brain cells, (iii) transcytosis, nanoparticles with bound bioactives, which can undergo transcytosis through the endothelial cell monolayer and gain access to the brain capillaries, (iv) inhibition of the P-gp efflux transporter and coating nanoparticles with specific P-gp inhibitory substances, such as polysorbate 80 that may possibly inhibit the P-gp, (v) membrane permeabilization effect that involves solubilization of the endothelial cell lipids via the use of surfactants, which leads to membrane fluidization and enhanced permeability of the BBB, and (vi) disruption of the BBB, thus opening tight junctions located between endothelial cells of the BBB; nanoparticles may assist in permeation of bioactives through tight junctions [84]. Nanocarriers are colloidal systems that include polymeric micelles, nanoparticles, lipid nanocapsules, and liposomes among others. Many of these systems have been designed and developed for indications in various CNS disorders to enhance brain uptake of the CNS targeted bioactives via P-gp transporter activity modulation.

### 9.2. Surfactant-Polymer Nanoparticles Overcome P-gp-Mediated Drug Efflux

Nanoparticles improve the therapeutic efficacy of an encapsulated drug by increasing and prolonging the delivery of the drug within the cell. Aerosol OT-alginate nanoparticles were employed in evaluating their delivery capabilities in brain tumor cells overexpressing P-gp. Rhodamine 123 and doxorubicin were employed as model P-gp substrates. Uptake studies using Rhodamine loaded nanoparticles showed a substantial increase in drug accumulation in resistant cells. The energy-dependent nanoparticle accumulation within cells proposes the contribution endocytosis in nanoparticle uptake. Nanoparticle doses of greater than 200 *μ*g/mL were shown to be the minimum concentration to improve drug accumulation [85, 86].

### 9.3. Lipid-Based Nanoparticles

**Figure 7:**
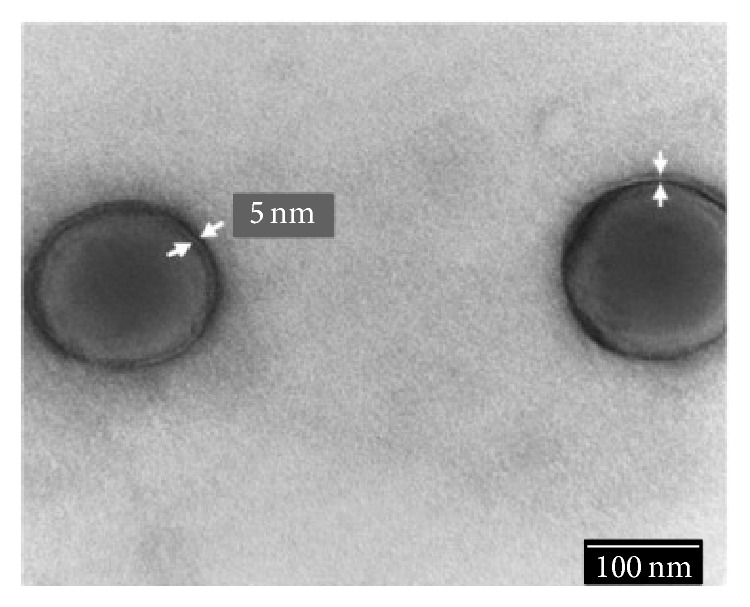
TEM images of lipid based nanoparticles; arrows show the lipid bilayer thickness [87] (reproduced with permission from Elsevier B.V. Ltd. © 2013).

Paclitaxel loaded lipid nanoparticles (Figure 7) utilizing Brij 78 displayed IC_50_ values 9-fold greater than when formulated with taxol. The study established that lipid nanoparticles were able to inhibit P-gp due to the presence of Brig 78, which was utilized in the synthesis of the microemulsion precursor. Temporary and reversible reductions in ATP were observed with blank nanoparticles and free Brig 78. Therefore, the greater degree of P-gp substrate accumulation was elucidated by a synergistic combination of nanoparticle with Brig 78, where nanoparticles improve the brain uptake of the bioactive by partially bypassing the P-gp transporter and drug efflux is restricted via the release of Brig 78 from the nanoparticles [50, 62, 87].

In other studies, lipid nanocapsules loaded etoposide were synthesized due to their hypothesized reversal of multidrug resistance by the use of P-gp inhibitory surfactants during formulation. P-gp inhibitory activity of the etoposide loaded lipid nanocapsules was independent of size. The proposed mechanism was cell uptake followed by intracellular P-gp inhibition thus allowing for a higher intracellular drug concentration [88]. In a further research study the formulation of lipid nanocapsules was achieved by the use of tricaprylic acid, polyethylene glycol 660, and soybean lecithin, for the delivery of the therapeutic agent, paclitaxel, to caco-2 cells; results showed that the oral bioavailability of paclitaxel was greatly improved possibly due to the ability of lipid nanocapsules to inhibit the P-gp efflux activity; this mechanism is primarily due to the fact that the surface layer of nanocapsules being phospholipids thus resulting in P-gp inhibition via P-gp substrate site competition [89].

### 9.4. Transferrin-Conjugated Nanoparticles for Delivery across the BBB

Transferrin-conjugated nanoparticles of poly(lactide)-D-*α*-Tocopheryl polyethylene glycol succinate diblock copolymer were formulated via the nanoprecipitation method with encapsulated docotaxel. Results from the study showed via IC_50_ data that transferrin-conjugated nanoparticles were 23.4–229% more efficient than the control, PLGA nanoparticles. Thus based on results obtained transferrin-conjugated nanoparticles showed to be effective in delivery of drugs across the BBB [90].

### 9.5. Paclitaxel-Polymer Micelles to Overcome Multidrug Resistance (MDR)

**Figure 8:**
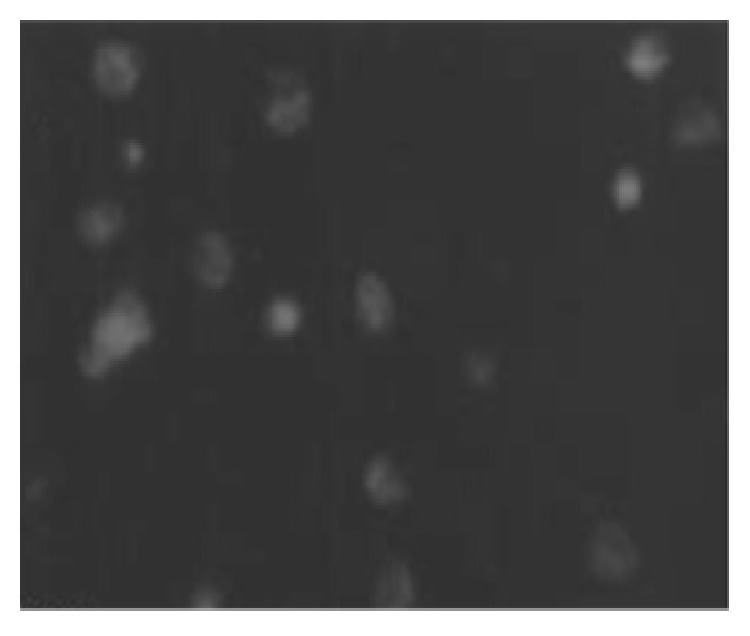
TEM micrographs showing the surface morphology of drug loaded micelles [91] (reproduced with permission from Elsevier B.V. Ltd. © 2011).

Micelles form from the spontaneous association of amphiphilic copolymers in aqueous phase and are characterized by a diameter no greater than 100 nm (Figure 8). The attractive force that leads to micellization is based on the interaction between the electrostatically neutral and hydrophobic portions of the copolymer. Self-assembly of the micelle only begins when the copolymer concentration reaches a threshold value generally referred to as the critical micelle concentration (CMC) [91]. Verapamil is a known P-gp inhibitor as well a reversal agent used in reversal of resistance associated with P-gp. Within the context of this study, paclitaxel, a P-gp substrate, in combination with verapamil, was encapsulated into micelles utilizing DOMC-FA (deoxycholic acid methylated chitosan-Folic acid). Resulting data showed that the conjugation of folate on to micelles displayed a higher brain tumor cell uptake of the loaded micelles, possibly via folate receptor-mediated endocytosis. Micelles consisting of the combination of verapamil and paclitaxel showed a higher cellular accumulation of paclitaxel as compared to micelles lacking verapamil [92].

In another study, mixed micelles of hydrophobic and hydrophilic pluronic L61 and F127 with encapsulated doxorubicin were formulated. Results observed depicted elevated cellular concentrations of drug to P-gp inhibition displayed via modification of intracellular drug transport [86].

### 9.6. Polyethylene Glycol-660 Hydroxystearate Nanocapsules

Nanocapsules encapsulated with etoposide (substrate) in combination with P-gp inhibiting surfactants, namely, PEG-HS (polyethylene glycol-660 hydroxystearate), were formulated with results depicting an increased bioavailability of etoposide [89, 93]. Further studies showed that lipid nanocapsules of paclitaxel formulated by triglycerides of capric and caprylic acids increased the uptake of drug molecules by MDR1 cells; the half-life of paclitaxel was also prolonged from 21 minutes initially to 5 hours [83].

### 9.7. Immunoliposomes in Bypassing P-gp

**Figure 9:**
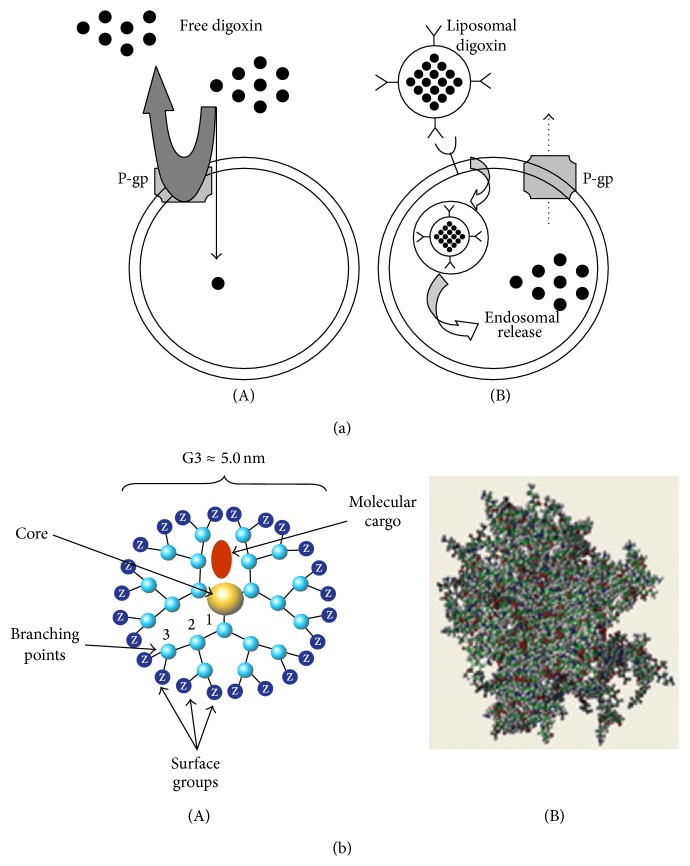
(a) (A) P-gp interacts with substrates in the plasma membrane and digoxin (substrate) is effluxed from the lipid bi-layer. (B) Cellular uptake of digoxin facilitated by Immunoliposomes-targeted to transferrin receptor and taken up via receptor-mediated endocytosis [94] (reproduced with permission from Drug Targeting Ltd. 2002). (b) (A) 2D Schematic representation of a dendrimer. (B) 3D representation of a dendrimer [100] (reproduced with permission from Elsevier B.V. Ltd. © 2009).

The overexpression of P-gp has been associated with the development of multidrug resistance in cancer cells. Methods employed in overcoming the multidrug resistance frequently involve the coadministration of inhibitors of the P-gp transporter (Figure 9(a)). Within the context of this study, the hypothesis that immunoliposome-based drug delivery systems may be employed as alternatives to overcome multidrug resistance has been explored. Since immunoliposomes penetrate target cells via receptor-mediated endocytosis, which in turn allows for the bypass of membrane related P-gp transporters, targeting of immunoliposomes was accomplished by via the use of an antitransferrin receptor monoclonal antibody (OX26 mAb). The incorporation of radiolabelled digoxin and OX26-immunoliposomes improved the cellular uptake of digoxin by a factor of 25 in immortalized rat brain endothelial cells. The cellular uptake as well as intracellular accumulation of the digoxin loaded liposomes was determined by acid washing of the cells and further confirmed via confocal microscopy studies. This* in vitro* study proposes that immunoliposome based drug delivery systems can be utilized to bypass P-gp and therefore deliver bioactives to the cytosol of target cells [94]. Furthermore there is the novel approach of combining substrates and P-gp inhibitors within liposomal delivery systems; clinical trials were performed via the formulation of liposomes encapsulating PSC 8339, a recognized P-gp efflux inhibitor in combination with the drug molecule, doxorubicin; results observed depicted an increase in drug delivery potential with no alteration to the pharmacokinetic or pharmacodynamic character of the drug molecule [95, 96].

### 9.8. PAMAM-Drug Complex for Delivery across the BBB

Dendrimers are defined as synthetic macromolecules that have a well-defined structural configuration. It is made up of a centrally located core and branched units that stem from the core (Figure 9(b)) [66, 97]. The mechanism by which dendrimers inhibit P-gp is via endocytosis; the dendrimer passes into the cell interior; this inhibition process could be due to cell membrane alterations and ATPase inhibition; research has shown that P-gp substrates that are encapsulated within dendrimers show an increased intracellular concentration [61]. Polyamidoamine (PAMAM) dendrimers belong to a new avenue of P-gp inhibition research; these dendrimers are known to improve the solubility of low solubility drugs. In the current study doxorubicin was chosen as the bioactive agent. Polyamidoamine (PAMAM) dendrimers were exploited as an effective carrier of doxorubicin. Data obtained from the fluorescence intensity assay and fluorescent microscopy displayed that cellular uptake of the PAMAM-doxorubicin complex was 6-fold greater than that of free doxorubicin. These results provide evidence that the novel PAMAM-bioactive complex is a simple yet highly efficacious system, with a great capability to cross the BBB and thus delivery CNS targeted drug successfully [98].

Propanolol, a P-gp substrate, was encapsulated within PAMAM dendrimers, due to its poor solubility. Conjugation of propanolol with PAMAM dendrimers was found to allow for bypass of the P-gp efflux pump and therefore enhance the drug concentration within target cells [99, 100].

### 9.9. Poly(D,L-lactide) Nanosuspensions of Risperidone

Nanosuspensions are dispersed systems comprising fine particles of drug molecules in the form of crystals. Nanosuspensions are known commonly to incorporate highly lipophilic compounds; these therapeutic agents are stabilized in solution via the use of surfactants. The large surface area afforded by the fine particles assists in enhancing bioavailability to the brain tissue, atovaquone coated by polysorbates in the form of a nanosuspension increased activity in mice presenting with encephalitis [83]. Employing Pluronic F-68 (P-gp inhibitor) nanosuspensions were formulated containing risperidone and poly-lactide; they have been found to display better clinical outcomes in psychotic disorders via P-gp inhibition [101].

### 9.10. Natural Vesicular Exosomes as Drug Delivery Systems to Possibly Bypass P-gp Efflux

Extracellular vesicles are defined as cell derived membrane vesicles with a characteristic (phospho) lipid bilayer that allows for cell to cell communication. Microvesicles are sized between 50 and 2000 nm, whereas exosomes are between 40 and 150 nm. Exosomes are produced by the formation of intraluminal vesicles in multivesicular bodies. Fusion of multivesicular bodies with the plasma membrane results in secretion of intraluminal vesicles which are in turn referred to as exosomes once released into the extracellular space. Due to their respective sizes and natural functionality, microvesicles and exosomes seem to be model candidates for drug delivery [102]. A study conducted by van Dommelen and coworkers provided the initial evidence of biotechnological modification of vesicular exosomes; immature dendritic cells was derived from mouse bone marrow as a source of exosomes. These were then loaded with exogenous siRNA for delivery. The BBB was chosen as the target site due to it being a vital obstacle to the delivery of drugs to the CNS. The study displayed specified delivery of siRNA to neurons in the brain with no corresponding toxicity. In another study performed, exosomes were used to deliver anti-inflammatory drugs to the brain via the intranasal route. It was proven that exosomes administered in this manner are potential delivery vehicles for small molecules, by enhancing biological stability and passage across the BBB; these systems may be extended to include a wider range of therapeutic agents [103].

Findings from research studies conducted thus far indicate that a vesicular exosomal-based formulation could be a valuable implement in future for the treatment of neurodegenerative disorders [104]. A comprehensive summary of the various drug delivery systems, polymers, and drugs employed in their formulation process and their respective applications is provided in Table 4.

## 10. Conclusions

Central nervous system (CNS) disorders such as schizophrenia are becoming increasingly prevalent, with limitations in social and physical functionality of patients being reduced. Treatment outcomes associated with schizophrenia therapy are reduced due to pharmacoresistance, which leads to a reduction in patient compliance. The P-glycoprotein (P-gp) efflux transporter located within the blood-brain barrier restricts the uptake of drugs and other molecules within the CNS; furthermore it has been implicated in the occurrence of pharmacoresistant schizophrenia. Therefore, research studies conducted in regards to the role of the P-gp transporter in CNS drug delivery prove to be a noteworthy avenue so as to improve schizophrenia treatment outcomes. The investigation of pharmaceuticals and novel drug delivery systems performed within the context of other CNS related disease states as reviewed has a promising applicability to the future therapy of pharmacoresistant schizophrenia due to P-gp efflux activity. Although P-gp modulating chemosensitizers show a noted reduction in P-gp function the use of high doses to achieve such an effect becomes a limiting factor to their use, due to toxic side effects. Conversely, the use of natural and synthetic polymers demonstrates highly efficient P-gp modulatory activity coupled with a pronounced safety profile and low cost; thus it is evident that these agents have a vast functionality in P-gp modulating systems. In addition a great amount of research has been focused on the use of nanotechnology in the bypassing of P-gp to allow for enhanced brain uptake of bioactive agents, with majority of these systems displaying success. However, the design and development of nanosystems are both time consuming and costly. Therefore, foreseen future trends lay in the benefit of applying the reviewed P-gp modulating agents into conventional delivery systems such as matrix tablets, which offer the added advantages of ease of administration, cost effectiveness, and improved patient compliance to treatment plans as it is a conventional method of drug delivery with a wide array of applications. Moreover, the need for P-gp modulating systems in schizophrenia treatment is vital, yet there remains a research gap in the development of drug delivery systems to combat P-gp efflux of antipsychotic agents at both the intestinal and BBB levels. Thus this review points to the various methods that may be applied in future schizophrenia drug delivery strategies.

## Figures and Tables

**Table 1:** The net uptake of bioactive by the brain depends on the difference between the influx and efflux processes [38] (reproduced with permission from Elsevier B.V. Ltd. © 2002).

Blood lumen	Endothelial cell	Extracellular fluid
(i) Blood Systemic exposure	$\overset{\;Influx\;\;clearance\;}{\to }$	
(ii) Drug blood cell and protein binding	(i) Drug permeability	
	(ii) Drug metabolism	Drug disposition
	$\overset{\;Efflux\;\;clearance\;}{\leftarrow }$	

**Table 2:** Brief overview of P-gp inhibitors/substrates and the corresponding IC_50_ values of inhibitors.

Inhibitor	IC_50_	References
Haloperidol	Potent	[105]

Fluoxetine Chlorpromazine Paroxetine Sertraline	Moderate	[59, 105]

Risperidone 9-hydroxyrisperidone Sulpiride Zolpidem	Low	[59, 105]

Loperamide^*∗*^		[59]

Verapamil^*∗*^ Quinidine^*∗*^	Moderate	[58, 59]

Prednisone^*∗*^	Low	[59]

Potent (P) inhibition (IC_50_ < 10 *μ*M), moderate (M) inhibition (10 *μ*M ≤ IC_50_ < 50 *μ*M), and low (L) inhibition (IC_50_ ≥ 50 *μ*M). ^*∗*^Non-CNS Inhibitors.

**Table 3:** Summarized list of polymer & surfactant P-gp inhibitors and their mechanism of action.

Polymer and surfactant based P-gp efflux inhibitors	Applications	References
*Natural polymers*:		
Xanthan gum Gellan gum Alginates	Inhibition of the P-gp efflux transporter by the presence of polysaccharide d-mannose monomers, increasing concentration of substrates, for example, vinblastine and doxorubicin.	[65]

*Synthetic polymers*:		
PEG 400 PEG-PEI PEG 300	Changes the microenvironment of Caco-2 cell membranes, leading to modifications in membrane fluidity.	[65, 66]

*Pluronic P85 *	Results in ATPase inhibition and ATPase reduction, as well as membrane fluidization.	[66, 68, 69]

*Thiomers *	Thiol groups interact with cysteine in the P-gp transmembrane channel, forming disulfide bonds. Thus the allosteric change blocks efflux.	[71]

**Table 4:** 

Drug delivery system	Polymer	Drug	Application	References
Nanocarrier (nanospheres)	Polyethylene glycol Polybutylcyanoacrylate, alginate, polyethylene glycol, polylactic –co-glycolic acid, and polylactic acid.	Doxorubicin, clozapine, methotrexate, saquinavir, and zidovudine	Drug molecule is delivered to the cell nucleus and thus avoids recognition by the BBB and intestinal P-gp efflux pump.	[83, 106–108]

Liposomes	Phospholipids and cholesterol	Epirubicin, cisplatin, methotrexate, and doxorubicin	It inhibits P-gp efflux transporter by interaction with phospholipids; the system may also incorporate P-gp inhibitors.	[95, 96]

Polymer nanoparticles	Acrylic polymers such as poly-butyl-cyanoacrylate (PBCA)	Loperamide and doxorubicin	It bypasses the P-gp due it its nanosize.	[106]

Dendrimers	Mannosylated poly(propyl eneimine) and polyethylene glycol-poly(amidoamine)	Vinblastine, doxorubicin, and propranolol (intestinal)	It inhibits P-gp efflux by bypassing the pump	[97–99]

Nanogels	Cationic and non-ionic polymers, NVP/NIPAM	Antisense phosphorothioate oligonucleotides (SODN) and 5-fluorouracil	It allows for the incorporation of P-gp inhibitors. Polysorbate coating of nanogel increases brain accumulation from 0.18 to 0.52%.	[83, 109]

Micelles	Pluronics L61 and F127 and P85	Doxorubicin, digoxin, paclitaxel, ritonavir, and vinblastine	(1) It inhibits P-gp efflux and also involves intracellular drug transport modification thereby avoiding the P-gp efflux pump. (2) Drug permeability in monolayered BBB model by P-gp substrates increased from 1.6- to 29-fold. No change in digoxin concentration in mdr1 knockout mice.	[83, 110, 111]

Hydrogels	(hydroxypropyl) Methacrylamide	Cyclosporine A and doxorubicin	It inhibits the P-gp efflux transporter by incorporation of drugs such as cyclosporine A that are P-gp inhibitors.	[112]

Lipid Nanocapsule	PEG-HS (polyethylene glycol-660 hydroxystearate), poly-methacrylate polymers, and HPMC	Etoposide and tacrolimus	(1) It inhibits P-gp efflux by incorporation of surfactant based P-gp inhibitors.	[83, 89]
	Triglycerides of capric and caprylic acids, Solutol	Paclitaxel	(2) Paclitaxel uptake increased in MDR1 expressing cells; the half-life in brain prolonged from 21 min to >5 h.	

Self-emulsifying drug delivery systems	Poly-methacrylate polymers	Paclitaxel and yacrolimus	It inhibits P-gp efflux by incorporation of surfactant based P-gp inhibitors, for example, Tween.	[113, 114]
	Medium chain triglyceride, PEG 400, polysorbate 80, and cremophor EL	Penfluridol (schizophrenia therapeutic agent)	It enhances solubilization of drug and thus effective brain concentrations.	
	Capmul MCM-C8, cremophor EL, and pluronic L-121	Irinotecan	It increased oral bioavailability by P-gp modulation.	

Nanosuspension	Pluronic F68	Risperidone	P-gp efflux inhibition by employing polymers that inhibit the P-gp transporter, such as pluronic.	[101]
